# Anthropogenic water sources and the effects on Sonoran Desert small mammal communities

**DOI:** 10.7717/peerj.4003

**Published:** 2017-11-10

**Authors:** Aaron B. Switalski, Heather L. Bateman

**Affiliations:** 1 College of Integrative Sciences and Arts, Arizona State University, Mesa, AZ, United States of America; 2 Cecil D. Andrus Wildlife Management Area, Idaho Department of Fish & Game, Cambridge, ID, United States of America

**Keywords:** Habitat structure, Developed waters, Plants, Wildlife waters, Military lands, Rodent, Arid ecosystems, Rodentia, Arizona, Species-habitat models

## Abstract

Anthropogenic water sources (AWS) are developed water sources used as a management tool for desert wildlife species. Studies documenting the effects of AWS are often focused on game species; whereas, the effects on non-target wildlife are less understood. We used live trapping techniques to investigate rodent abundance, biomass, and diversity metrics near AWS and paired control sites; we sampled vegetation to determine rodent-habitat associations in the Sauceda Mountains of the Sonoran Desert in Arizona. A total of 370 individual mammals representing three genera and eight species were captured in 4,800 trap nights from winter 2011 to spring 2012. A multi-response permutation procedure was used to identify differences in small mammal community abundance and biomass by season and treatment. Rodent abundance, biomass, and richness were greater at AWS compared to control sites. Patterns of abundance and biomass were driven by the desert pocket mouse (*Chaetodipus penicillatus*) which was the most common capture and two times more numerous at AWS compared to controls. Vegetation characteristics, explored using principal components analysis, were similar between AWS and controls. Two species that prefer vegetation structure, Bailey’s pocket mouse (*C. baileyi*) and white-throated woodrat (*Neotoma albigula)*, had greater abundances and biomass near AWS and were associated with habitat having high cactus density. Although small mammals do not drink free-water, perhaps higher abundances of some species of desert rodents at AWS could be related to artificial structure associated with construction or other resources. Compared to the 30-year average of precipitation for the area, the period of our study occurred during a dry winter. During dry periods, perhaps AWS provide resources to rodents related to moisture.

## Introduction

Water is seen as a limiting resource for the distribution of many animal species in arid environments (Roberts, 1977; Broyles, 1997; Rosenstock, Ballard & Devos, 1999). Within western North America, supplemental water has been used as a management tool for game species and livestock (Broyles, 1997) and for mitigating the loss of natural water sources from increased aridity, human use, and urbanization (Dolan, 2006; Longshore, Lowery & Thompson, 2009). Natural resource managers commonly use anthropogenic water sources (AWS, e.g., guzzlers, stock tanks, earthen ponds, and other constructed water sources) to supplement or enhance existing natural sources of water in arid environments (Krausman, Rosenstock & Cain, 2006).

Despite the frequency of AWS construction in arid ecosystems, little is known about how AWS influence wildlife (Broyles, 1995; Kluever, Gese & Dempsey, 2017). Studies have investigated the population dynamics of game species in ranges with and without AWS (Broyles & Cutler, 1999 showing no effect of AWS) and species interactions among predators visiting AWS (DeStefano, Schmidt & DeVos, 2000; Atwood, Fry & Lelane, 2011; Brawata & Neeman, 2011; Hall et al., 2013). Researchers have questioned whether free water from AWS is beneficial or harmful to species adapted to arid systems (Burkett & Thompson, 1994; Cain III et al., 2008; Griffis-Kyle, Kovatch & Bradatan, 2014). However, the effects of AWS on non-game species (Simpson, Stewart & Bleich, 2011) are understudied.

Research focused on nongame species’ use of AWS has found similar abundances and richness of small mammals at AWS compared to non-AWS (Cutler & Morrison, 1998). In contrast, Burkett & Thompson (1994) documented higher abundances of small mammals at AWS but concluded there was no biological linkage to the presence of water and greater mammal abundances were related to ground disturbance and construction debris in the vicinity of AWS. Because AWS can include constructed materials (e.g., wood piles, concrete, and sheet metal) and clearing vegetation, there is justification to investigate how vegetation structure surrounding AWS has been modified and might influence the abundance of non-target species such as rodents.

Desert rodents are one of the most studied animal communities in the American Southwest (Heske, Brown & Mistry, 1994; Valone & Brown, 1995; Valone & Saunter, 2005; Thibault et al., 2010). Rodent communities are affected by many processes (e.g., disturbance, predation) and are sensitive to changes in habitat structure (Whitford, 1997; Valone & Saunter, 2005). In the southwestern US, some species of rodents are associated with sandy soils and creosote bush (*Larrea tridentata*; Bailey, 1931; Price, 1984) and rodents such as woodrats (*Neotoma* sp.) prefer areas with high density of cacti (Spencer & Spencer, 1941) and human-modified habitats (Chamblin, Wood & Edwards, 2004). Burrowing rodents are considered ecosystem engineers because they affect vertebrate and plant composition (Davidson & Lightfoot, 2006; Davidson, Lightfoot & McIntyre, 2008). Because of their abundance, well-documented ecology, and ecological importance in arid environments, small mammal communities may be used as a model to better understand the indirect effects of AWS on non-game species in arid lands.

The goal of this study was to investigate the effects of AWS on desert rodent communities. Our research objectives were to compare AWS sites and non-AWS control sites to determine (1) how sites varied in rodent species abundance, biomass, richness, and diversity, (2) how vegetation and habitat structure varied, and (3) which vegetation characteristics predicted small mammal species’ occurrence.

## Methods

### Study area

We conducted this study within the Sauceda Mountains on the Barry M. Goldwater Range (BMGR-East), a 424,919 ha military training area 39 kilometers south of Gila Bend, in Arizona, USA. Multiple AWS were constructed to support desert bighorn sheep (*Ovis canadensis nelsoni*) populations and draw federally endangered Sonoran pronghorn (*Antilocapra americana sonoriensis*) away from military testing ranges. The presence of AWS and relatively undisturbed Sonoran Desert make BMGR-East an ideal site to investigate effects of AWS on vegetative structure and rodent communities. Study site elevations ranged from 425 to 730 m. Topography was characterized by large hills, valleys, and ephemeral washes with vegetation characteristic of Arizona Upland and Lower Colorado subdivisions of the Sonoran Desert scrub community (Brown & Lowe, 1980). Study sites within these two subdivisions and ephemeral washes supported plant species including, creosote bush (*Larrea tridentata*), triangular bursage (*Ambrosia deltoidea*), yellow paloverde (*Parkinsonia microphylla*), saguaro cactus (*Carnegiea gigantean*), cholla (*Cylindroptunia* spp.), *Acacia* spp, and ocotillo (*Fouquieria splendens*). Additional species present in xeroriparian areas were thornbush (*Lycium* spp.), velvet mesquite (*Prosopis velutina*), desert ironwood (*Olneya testoa*), and desert honeysuckle (*Anisacanthus thuberi*). Special use permit #2012-01 was issued for access onto the BMGR-East by the 56th Range Management Office of Luke Air Force Base, Arizona.

### Mammal trapping

**Figure A1:**
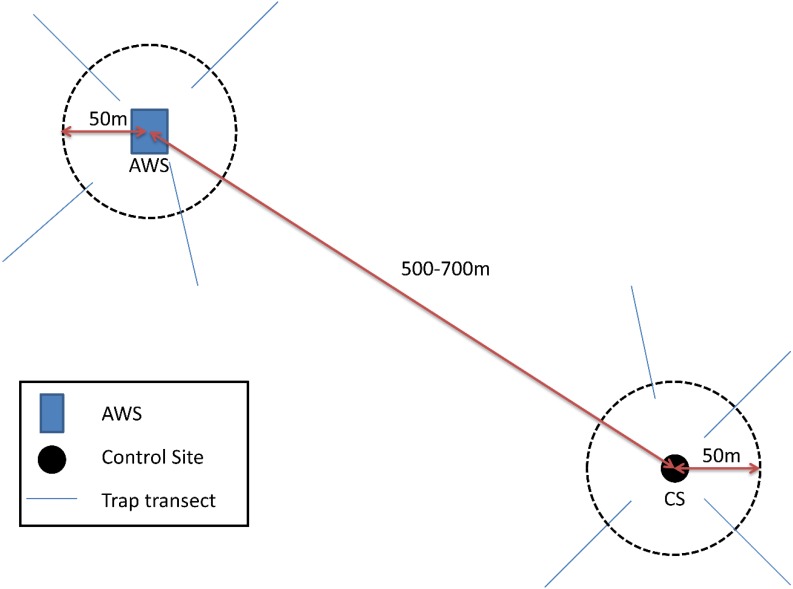
Configuration of randomly placed trapping transects around anthropogenic water sources (AWS) and control sites (CS) to sample small mammal communities during winter 2011 and spring 2012 on Barry M. Goldwater Range in Maricopa County, Arizona, USA. Control sites originated from random points generated in GIS and located 500–700 m from AWS. Transect starting locations were randomized, as were the orientation or bearing of transects, and placed within 50 m of AWS or CS.

To determine differences in mammal abundance, biomass, richness, and diversity at AWS and non-AWS control sites, we live-trapped rodents during winter 2011 (October–January) and during spring 2012 (February–May). Because maximum daily temperatures exceed 41 °C during the summer (30-year average, NOAA weather station USC00023393 at Gila Bend, AZ), we trapped during cooler periods to reduce heat-stress on animals. We trapped rodents during four sessions (two sessions per season), with three trap-nights per session. Rodents were trapped at five AWS including four human-constructed sites and one modified natural site (tinaja—depression formed in bedrock carved by rainfall or seepage). Rodents were trapped at five control sites from the surrounding desert. Trapping sessions 1 and 2 during winter averaged 54 days apart; trapping sessions 3 and 4 during spring averaged 48 days apart. Trapping sessions between seasons (sessions 2 and 3) averaged 94 days apart. We trapped along 135 m transects with 10 traps per line. We used Sherman live traps (five traps of 8 × 9 × 23 cm and five traps of 8 × 9 × 33 cm) and alternated trap type along the transect (*sensu* Burkett & Thompson, 1994; Pearson & Ruggerio, 2003; Hopkins & Kennedy, 2004). Each site (AWS and CS) had four trapping transects placed randomly using ESRI ArcMAP 10 software (Environmental Systems Research Institute, Redlands, CA, USA). For example at AWS, we generated four random points within 50 m of the tank or tinaja and transects radiated away on random bearings (1–360) that did not intersect with other transects around the AWS (Fig. A1). Control sites were selected by generating random points using ArcMAP that occurred between 500 and 700 m from each AWS (Fig. A1). The orientation of control transects were also selected by choosing a random bearing. Transects were not revisited and new transects were established during each trapping session. Therefore, the sample size to estimate mammal abundance, biomass, richness, and diversity was *n* = 80 transects during winter and *n* = 80 transects during spring (i.e., 5 sites × 4 transects × 2 treatments × 2 sessions = 80).

Traps were placed before sundown and baited with apple wafer pellets (Manna Pro; St. Louis, MO, USA). Traps were checked starting 1.5 h before sunrise each day unless stormy weather and rain warranted checking traps earlier. Polyester or cotton batting was placed in each trap during winter trap sessions to reduce exposure and minimize trap mortality. Captured animals were identified to species (Hoffmeister, 1986), aged (e.g., juvenile or adult), sexed, and weighed. Animals were individually marked with self-piercing metal tags applied to ear pinnae using an applicator (National Band and Tag Company, New Port, KY, USA) in the left ear. We also marked ears with permanent ink marks, with individually unique patterns of our own design, in case tags tore from pinnae. Animals were released at their site of capture. Animals were handled and processed following Arizona State University Institutional Animal Care and Use Committee (IACUC) protocol #09-1051R.

### Vegetation sampling

**Figure 1:**
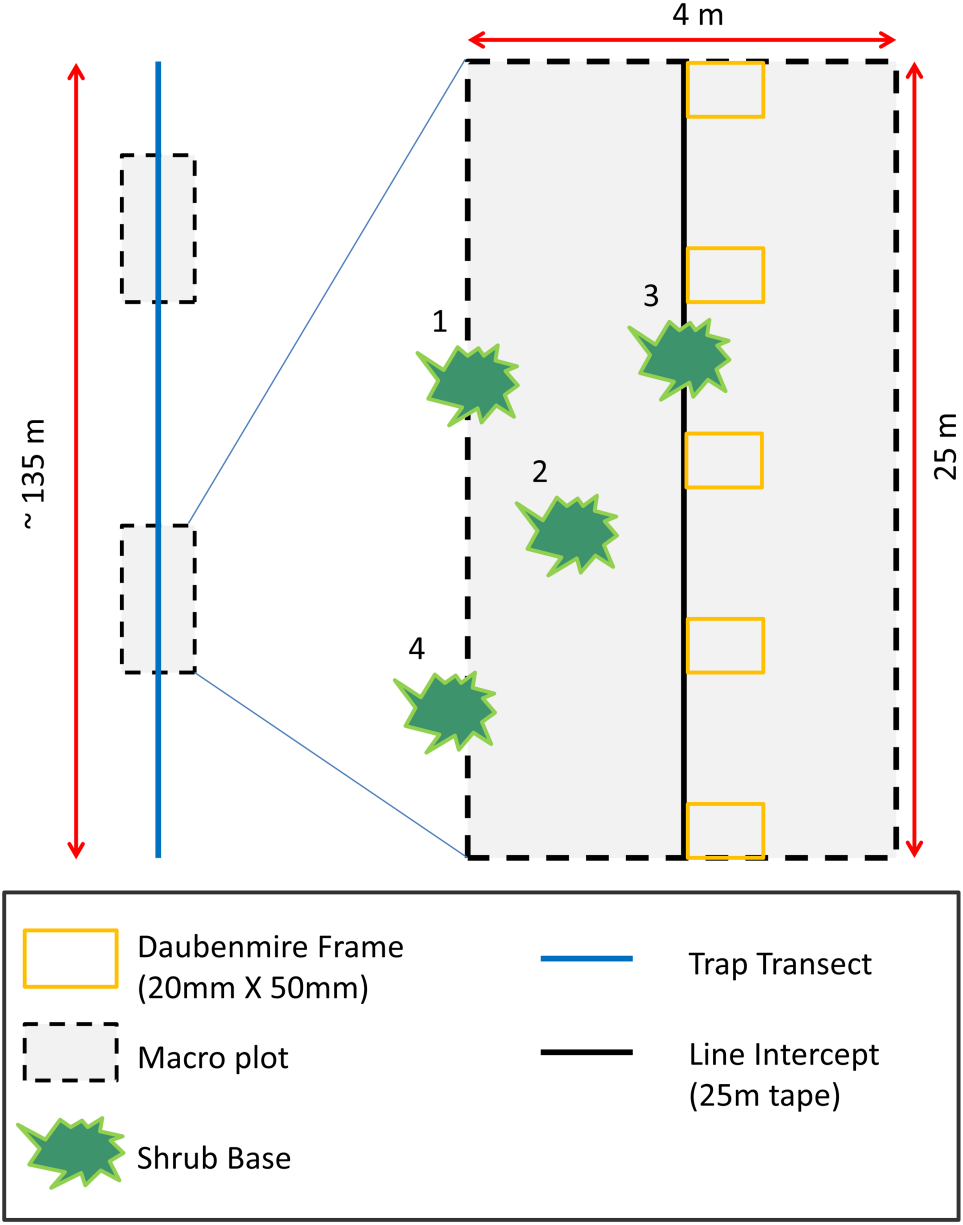
Habitat sampling diagram depicting placement of two macro-plots randomly located along trapping transects. Shrub and tree cover estimates were performed along the midline of 25 m long macro-plots using line intercept methods. Herbaceous cover was recorded in six Daubenmire frames placed every 5 m along the midline of macro-plot. Plant richness and density estimates were performed inside the macro-plot. Plants with bases ≥50 percent inside macro-plots were considered in the macro-plot (numbers 1–3 in diagram). Plants with bases <50 percent inside plots were considered outside the macro-plot (number 4 in diagram).

To assess how vegetation differed between AWS and CS and to relate vegetation to rodent occurrence, we measured plant species richness, density, and cover. To quantify plant density and species richness (Epple & Epple, 1995), we collected data during the spring season in two 4 × 25 m (0.01 ha) macro-plots randomly located along each trapping transect (Carpenter, 1999; Fig. 1). We recorded shrub cover along the midline of macro-plots using line intercept sampling techniques (Canfield, 1941). We placed (Daubenmire, 1959) frames (20 × 50 mm) at 5 m intervals along the line intercept to measure herbaceous cover. Grasses and forbs were estimated visually and placed into 12 cover classes (<1, 1–5, 6–15, 16–25, 26–35, 36–45, 46–55, 56–65, 66–75, 76–85, 86–95, >95%).

### Data analyses

We calculated relative rodent abundance (hereafter, abundance) as the number of unique individuals captured per 100 trap nights. Analyses were done at the level of the transect. We determined transects to be independent because transects did not overlap in space or time and we did not recapture marked animals across transects. Species richness was the average number of species captured per transects per treatment. Mammal biomass was calculated using the mean mass for each individual (if an individual was encountered more than once during a three-night trap session, mass was averaged) and then summing the mass for each species on each transect. Simpson’s diversity index (Simpson, 1949) was calculated to examine rodent diversity between treatments. Occurrence was determined as presence/absence of each species per transect. Where data did not meet assumptions of normality, we utilized a non-parametric multivariate analysis, called non-parametric multi-response permutation procedures (MRPP; Biondini, Mielke & Berry, 1988), to investigate differences in rodent community attributes (i.e., abundance, biomass, and species richness) between the two treatments (AWS and CS). A Sidak correction was utilized to adjust for type I error across multiple MRPP tests (Abdi, 2007).

We summarized variation in vegetation between treatments using a principal component analysis (PCA), using IBM SPSS version 20 (IBM Corp, Armonk, NY, USA). PCA is a multivariate technique to reduce many correlated independent variables into a set of uncorrelated axes called principal components (Legendre & Legendre, 1998). To interpret each component of the PCA, we considered vegetation variables that loaded high (>0.500) in the component matrix. We used eigenvalues and scree plots, which are explained variances, to discriminate the relative importance of each component. Principal component scores and vegetation variables were compared between treatments using a Mann–Whitney rank sum test.

To explain species-habitat relationships, we used species’ occurrences as the response variables because species were not ubiquitous in the study area. We correlated occurrence with principal component scores (predictor variables) using logistic regression (Legendre & Legendre, 1998). Because vegetation was only measured during the spring (and not during the winter) and we wanted to relate mammals captured along transects to where vegetation was sampled, we only used mammal capture data from the spring in habitat models (*n* = 80).

## Results

### Small mammals

During our study, we captured 370 individuals representing three genera and eight species of rodents across 4,800 total trap nights. The most common species encountered was the desert pocket mouse (*Chaetodipus penicillatus*, 198 captures). Other species encountered included the rock pocket mouse (*C. intermedius*, 67 captures), Bailey’s pocket mouse (*C. baileyi*, 42 captures), Merriam’s kangaroo rat (*Dipodomys merriami*, 28 captures), white-throated woodrat (*Neotoma albigula*, 22 captures), cactus mouse (*Peromyscus eremicus*, 11 captures), Arizona pocket mouse (*Perognathus amplus*, 1 capture), and Harris’s antelope squirrel (*Ammospermophilus harrisii,* 1 capture; Table A1). Cactus mouse, Arizona pocket mouse, and Harris’s antelope squirrel were excluded from species-habitat analyses due to their limited number of captures. The remaining five species represented more than 90 percent of captures and were included in analyses.

**Figure 2:**
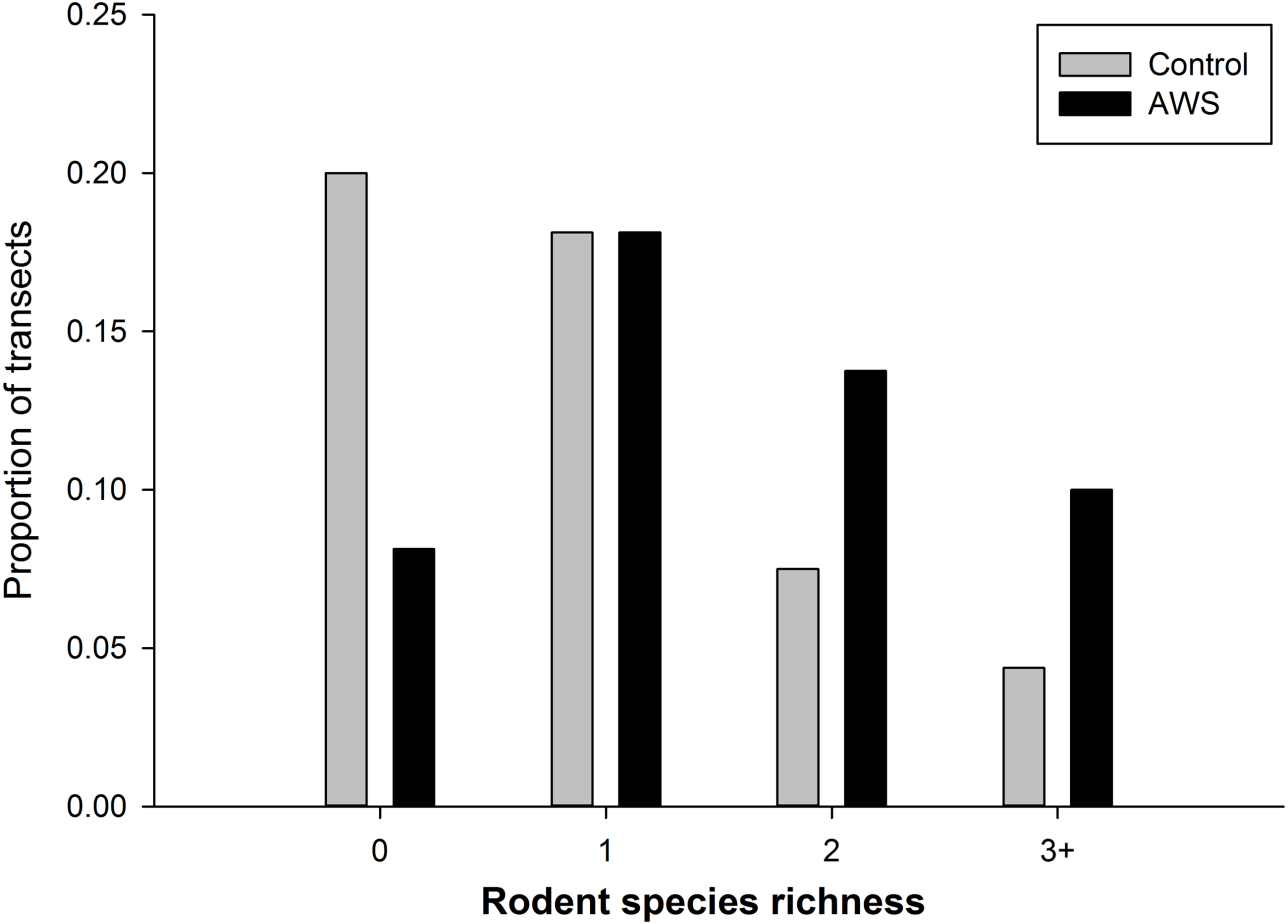
The frequency of the number of species of rodents that occurred at anthropogenic water sources (AWS) and control sites during winter 2011 and spring 2012 on the Barry M. Goldwater Range in Maricopa County, Arizona, USA.

We did not detect seasonal (winter vs spring) differences in rodent community attributes (standard errors in parentheses, *n* = 160). Rodent abundance was 8.9 individuals per 100 trap nights (±1.0) in winter and 6.4 individuals per 100 trap nights (±0.8) in spring (MRPP, *P* = 0.054). Rodent biomass was 63.9 g (±7.8) in winter and 62.4 g (±11.1) in spring (MRPP, *P* = 0.306). Richness was 1.3 species (±0.1) in winter and 1.2 species (±0.1) in spring (MRPP, *P* = 0.567). However rodent community abundance and biomass differed between treatments (MRPP, *P* < 0.001 for both metrics); with abundance almost twice as high at AWS compared to CS (Table 1). Rodent diversity was similar between treatments with Simpson diversity indices of AWS and CS equal to 2.859 and 2.971, respectively. Similar species were encountered at both treatments; however, richness per trapping transect was greater at AWS compared to CS (MRPP, *P* < 0.001; Table 1). Nearly 40% of CS transects encountered either no animals or only a single species (Fig. 2).

**Table 1:** Mean (±SE) rodent community variables during winter 2011 and spring 2012 at anthropogenic water source (AWS) sites and control sites (CS) on Barry M. Goldwater Range in Maricopa County, Arizona, USA. Abundance is the number of individuals captured per 100 trap nights. Biomass measured in grams is the sum of all individuals captured per species averaged per transect. Species richness is the average number of species captured per transects per treatment. Test statistics reported are for multi-response permutation procedure (MRPP); *n* = 160; *α* = 0.05.

Variable	AWS	CS	Statistic	*P*
Abundance	10.1 (0.6)	5.3 (0.7)	−9.5	<0.001
Biomass	89.6 (7.8)	40.4 (5.2)	−10.2	<0.001
Species richness	1.6 (0.1)	0.9 (0.1)	−8.0	<0.001

**Figure 3:**
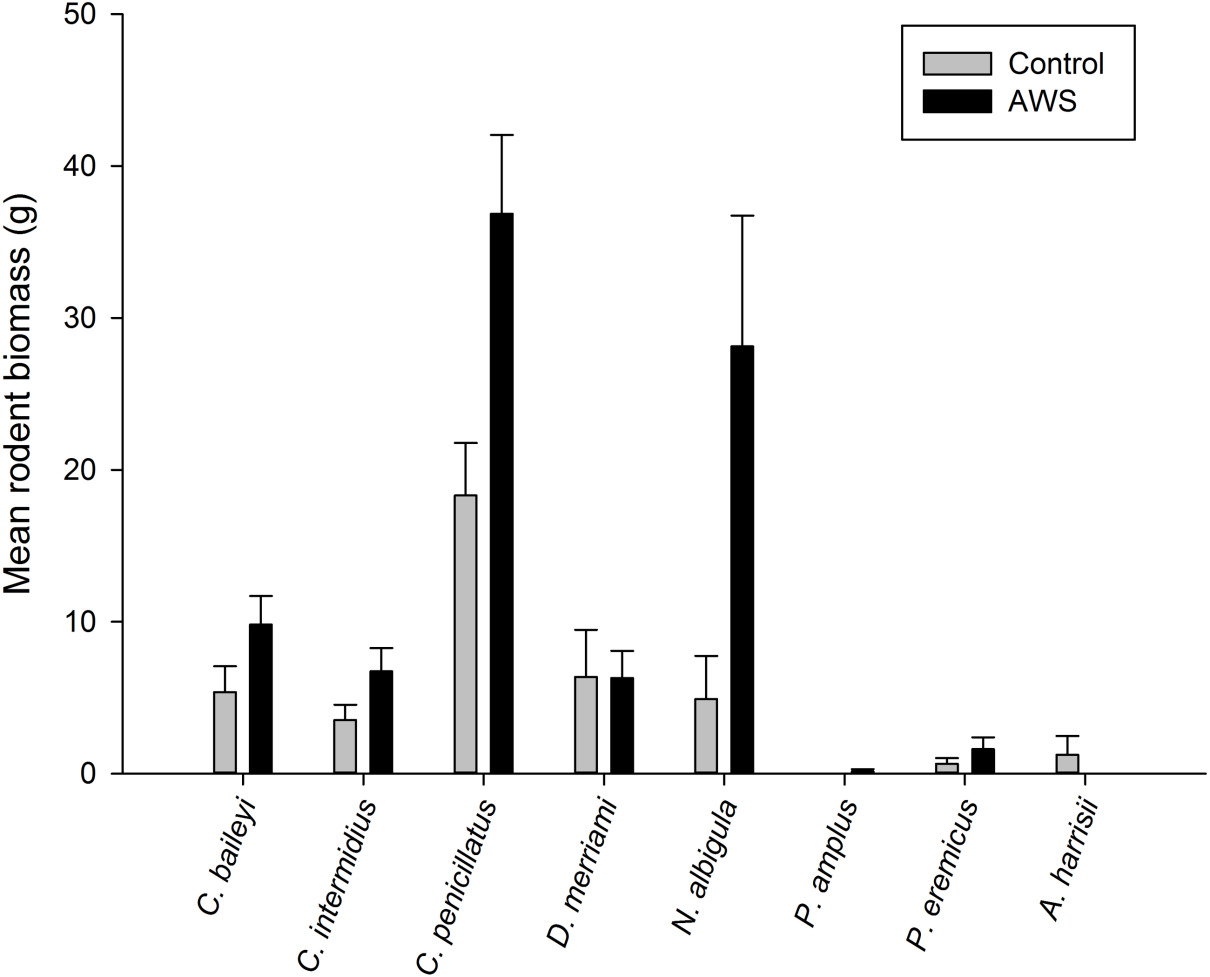
Mean (±SE) of rodent biomass (grams) at anthropogenic water sources (AWS) and control sites during winter 2011 and spring 2012 (during 4,800 trap nights, *n*= 16) on the Barry M. Goldwater Range in Maricopa County, Arizona, USA.

Only two rodent species showed differences in abundance and biomass between AWS and CS. Desert pocket mouse abundance was greater at AWS (Table 2) and biomass at AWS was nearly twice that of CS (Fig. 3). Biomass of white-throated woodrat was over five times greater at AWS (Fig. 3).

**Table 2:** Mean (±SE) number of individuals captured per 100 trap nights during winter 2011 and spring 2012 at anthropogenic water source (AWS) sites and control sites (CS) on Barry M. Goldwater Range in Maricopa County, Arizona, USA. Test statistics reported are for Multi-response Permutation Procedure (MRPP), *n* = 160 (5 tests, *α* = 0.05, Sidak correction = 0.010).

Family	AWS	CS	Statistic	*P*
Species				
Heteromyidae				
*Chaetodipus penicillatus*	5.5 (0.8)	2.8 (0.5)	−4.1	0.007
*Chaetodipus baileyi*	1.0 (0.2)	0.6 (0.2)	−2.9	0.023
*Chaetodipus intermedius*	1.9 (0.4)	1.0 (0.5)	−1.2	0.106
*Dipodomys merriami*	0.6 (0.2)	0.5 (0.2)	0.3	0.490
Cricetidae				
*Neotoma albigula*	0.7 (0.2)	0.2 (0.1)	−2.9	0.022

### Vegetation characteristics

We reduced 11 vegetation variables into five principal components which accounted for 91.1% of variation at the AWS and CS sites (Table A2). Variables associated with the presence of water (i.e., distance to AWS or distance to wash) did not explain a large percentage of variation and were not included in the final PCA. We interpreted principal component 1 as ground cover; principal component 2 as shrub cover; principal component 3 as tree overstory; principal component 4 as cactus density; and principal component 5 as shrub density (Table A2). Overall, AWS and CS were very similar in vegetation characteristics in terms of cover and vegetation density (Table 3).

**Table 3:** Mean (±SE) of vegetation variables and principal component (PC) values at anthropogenic water sources (AWS) and control sites (CS) during spring 2012 on Barry M. Goldwater Range in Maricopa County, Arizona, USA. Test statistics reported Mann–Whitney Rank Sum Test (*U*), *n* = 80. PCA correlation matrix reported in Table A2.

	AWS	CS	Statistic (*U*)	*P*
** Habitat variables**				
Bare ground (% cover)	93.0 (0.5)	93.0 (0.7)	764.0	0.733
Herbaceous (% cover)	7.4 (0.4)	7.5 (0.6)	764.5	0.736
Forbs (% cover)	6.8 (0.5)	6.6 (0.7)	728.5	0.494
*L. tridentata* (% cover)	6.6 (1.1)	6.4 (0.8)	773.0	0.798
Shrub (% cover)	13.0 (1.1)	12.4 (1.0)	777.0	0.829
*L. tridentate* density/ha.	296.3 (34.7)	351.2 (41.7)	720.0	0.443
Tree density/ha.	190.0 (37.1)	142.5 (21.1)	761.0	0.708
Tree (% cover)	6.3 (1.2)	4.8 (0.8)	754.0	0.658
*C. leptocaulis* density/ha.	48.7 (15.6)	123.6 (44.8)	742.5	0.515
Cacti density/ha.	293.8 (34.0)	436.2 (71.7)	661.5	0.183
Shrub density/ha.	2025.0 (175.1)	2005.0 (184.6)	780.0	0.851
** Principal components**				
(PC1) Ground cover	−0.022 (0.13)	0.022 (0.18)	772.0	0.791
(PC2) Shrub cover	−0.003 (0.17)	0.003 (0.14)	753.0	0.655
(PC3) Tree overstory	0.176 (0.02)	−0.176 (0.11)	687.0	0.279
(PC4) Cactus density	−0.215 (0.08)	0.215 (0.20)	619.0	0.082
(PC5) Shrub density	0.007 (0.15)	−0.007 (0.16)	792.0	0.942

Of the five species of rodents included in species-habitat analyses, three showed significant relationships with principal components (Table 4). Bailey’s pocket mouse occurrence was positively related to areas with higher cactus density (PC4). Merriam’s kangaroo rat occurrence was negatively related to areas with high tree and shrub density (PC5) and high tree cover (PC3). Rock pocket mouse occurrence was negatively influenced by greater herbaceous ground cover (PC1).

**Table 4:** Occurrence of rodent species predicted by vegetation characteristics (principal components, PC) from Principal Component Analysis using logistic regression (*n* = 80). Direction of correlation indicated by C for correlation. Test significance (*P*-values) and model fit (percent classification accuracy) are reported. Rodents were captured at anthropogenic water sources and control sites during spring 2012 on the Barry M. Goldwater range, Maricopa County, Arizona, USA.

Species	C	Habitat	Statistic	*P*
*Chaetodipus baileyi*	+	Cactus density (PC4)	5.0	0.024 (82.5%)
*Chaetodipus intermedius*	−	Ground cover (PC1)	7.1	0.008 (66.3%)
*Chaetodipus penicillatus*	+	Cactus density (PC4)	2.0	0.154 (55.0%)
*Dipodomys merriami*	−	Tree overstory (PC3)	12.2	0.002 (91.3%)
	−	Shrub density (PC5)		
*Neotoma albigula*	+	Cactus density (PC4)	1.6	0.201 (86.3%)

## Discussion

The effects of AWS on non-game species are not well studied, but our results suggest that rodent abundance and biomass were greater at AWS compared to CS in southern Arizona. The rodent community was dominated by habitat generalist species, such as the desert pocket mouse. Because of their large number of captures and body mass, desert pocket mouse and white-throated woodrat had the greatest influence on these parameters of total abundance and biomass. AWS had a similar species composition as CS but AWS had greater species richness.

Generally, we documented similar vegetation and structural characteristics around AWS and adjacent desert CS at BMGR-East. Some of differences in vegetation, such as lower creosote bush (*Larrea tridentata*) and cactus (*Cylindroptunia* spp.) densities around AWS, could be the result of vegetation clearing when an AWS was initially installed or renovated. It is typical for shrubs and cacti to be cleared or trans-located prior to AWS installation (Arizona Game and Fish Department, 2008). Although habitat models for the two most numerous species (in captures and in biomass) were not conclusive, we did find that three species of rodents were associated with elements of cover from cacti or avoided area without cover, such as areas with high amounts of herbaceous and grass cover.

Species-habitat relationships from this study were consistent with findings from other research. Merriam’s kangaroo rat occurrence was negatively related to high shrub and tree density. Merriam’s kangaroo rat is associated with open areas with few shrubs and trees (Rosenzweig & Winakur, 1969; Cutler & Morrison, 1998; Stevens & Tello, 2009) and found in areas without dense riparian vegetation (Bateman & Ostoja, 2012). In our study, rock pocket mouse occurrence was negatively associated with higher amounts of herbaceous ground cover and low amounts of bare ground. This finding was consistent with other descriptions of habitat use, with rock pocket mouse preferring rocky soils, bare ground, and areas with limited herbaceous growth (Hoover, Whitford & Flavill, 1977). Bailey’s pocket mouse and white-throated woodrat occurrence were positively related to higher densities of cactus. Similarly, Brown and colleagues found that desert woodrat (*Neotoma lepida*) density was dependent on the presence of teddy bear cholla (*Cylindroptunia bigelovii*) (Brown, Lieberman & Dengler, 1972). However, only Bailey’s pocket mouse relationship with cactus density was significant in our study. The desert pocket mouse is considered a habitat generalist associated with sandy soils and creosote bush (Price, 1984). The habitat models for this generalist species in our study were inconclusive. Implications for understanding which species are associated with specific elements of vegetation structure can help explain possible differences in abundance around AWS. For example, we found that some rodent species were associated with vegetation structure and, although vegetation characteristics did not vary between AWS and CS, perhaps structure might be substituted for artificial debris around AWS.

Perhaps, other factors related to structure may have contributed to differences in rodent communities between AWS and CS. Although not quantified during this study, soil disturbance and greater amounts of artificial structure (i.e., construction debris, above ground tanks, rain collectors) were observed at AWS compared to the surrounding desert. These human-constructed elements may have effected rodent abundance by providing structure preferred by some species. Burrowing species of rodents (i.e., Merriam’s kangaroo rat) favor disturbed soils with better burrowing conditions (Schmidly, Williams & Derr, 1988). Breck & Jenkins (1997) suggested that Merriam’s kangaroo rat were associated with sandy or loose soils because burrow and mound construction could have a lower energetic cost in disturbed soil. Burkett & Thompson (1994) suggested that debris and structure in the vicinity of AWS provided additional habitat for rodent species as a possible explanation for higher abundances near AWS.

One possibility is that rodents may have benefitted from access to moist microhabitats. Desert rodents, particularly the family Heteromyidae, have physiological adaptations (e.g., specialized kidneys, concentrated urine) and behavioral adaptations (e.g., torpor, burrowing and nocturnal activity) to minimize water loss and metabolize water from food instead of drink free water (Kenagy, 1973; MacMillen & Hinds, 1983; Franks, 1988). Merriam’s kangaroo rats can obtain water by caching seeds in humid burrows where the dry seeds take up moisture (Nagy, 1994). White-throated woodrats are similarly well equipped for survival in arid habitats by adaptations such as nocturnal activity and feeding on succulent fruits (i.e., cactus; Brown, Lieberman & Dengler, 1972). We determined that the three months of winter rainfall (Dec–Jan–Feb) during our study was 12.4 mm which was only 19% of 30-year average amounts of precipitation for the same time interval (NOAA weather station USC00023393 at Gila Bend, AZ, USA). We sampled within 50 m of an AWS, including one natural water catchment (tinaja); therefore, we could have encountered rodents that had access to areas near water. Perhaps, during this drier period, some species may have benefit from moister microhabitats.

Desert rodent abundance near AWS could have been influenced by supplemental food resources from tanks. Although the majority of species captured during our study were granivorous (i.e., Heteromyidae), previous studies have observed that species commonly accepted as granivorous do supplement their diet with succulent vegetation and insects (Hope & Parmenter, 2007). In New Mexico, Orr and colleagues documented seasonal use of arthropods in granivorous, heteromyid rodents from September to November and, to a lesser extent, May and June (Orr, Newsome & Wolf, 2015). Although we did not investigate the insect community, a study in southwestern Arizona by Griffis-Kyle and colleagues documented dragonfly use of AWS and found that natural tinajas had 2–3 species present (Griffis-Kyle, Kovatch & Bradatan, 2014). Perhaps insect resources around AWS might provide one possible explanation for our observation that rodent abundance and biomass was greater near AWS, particularly during a dry winter when forage species might be reduced.

It is important to note that other studies investigating small mammal communities in the vicinity of AWS documented mixed responses. Some researchers found that rodent abundances were higher at AWS when compared to areas without waters (Burkett & Thompson, 1994); whereas, Cutler & Morrison (1998) observed no difference in abundance at AWS. Additional studies of wildlife populations, seasonal dietary selection, of species-habitat relations before and after the installation of AWS could provide additional insight into the direct and indirect effect of AWS on wildlife.

### Management implications

The use of water as a management tool for endangered or game species is popular and has increased in recent history. Even with debate about its effectiveness as a management tool (Broyles, 1995) state, federal, and private agencies have allocated large sums of resources to install and maintain AWS. Over a decade ago, Arizona was spending $750,000 annually on AWS (Rosenstock, Ballard & Devos, 1999). The result of this study, even with a short sampling period and focus on organisms that experience fluctuating populations (Krebs & Myers, 1974), suggests that some species of wildlife may increase in abundance near AWS because of changes to vegetation, utilization of human-modified structures, or perhaps changes in food resources. Our trapping data may contribute to understanding patterns of small mammal use of AWS during a dry winter, that will likely become more common as the environment gets hotter and drier in the Southwest (Ye & Grimm, 2013). Combined with past research on AWS, our results will help managers make informed decisions about construction and maintenance of AWS as a management tool.

##  Supplemental Information

10.7717/peerj.4003/supp-1 Data S1 Raw data Raw data includes number and species of mammals captured, mass of individual mammals, vegetation measurements, and site coordinates.

## Appendix

**Table A1:** Mean (±SE) characteristics of rodent species captured in Sherman traps during winter 2011 and spring 2012 on Barry M. Goldwater Range in Maricopa County, Arizona, USA. Some individuals were not measured for all morphometrics (number of individuals measured in each category given by *n*).

Family	*n*	Body mass (g)	Body length (mm)	Hind foot length (mm)	Tail length (mm)
Species					
Heteromyidae					
*Dipodomys merriami*	38∕37∕32∕37	36.6 (0.9)	88.7 (1.1)	35.6 (0.3)	143.1 (1.2)
*Chaetodipus penicillatus*	101∕102∕89∕102	22.5 (0.3)	73.6 (0.6)	24.5 (0.1)	109.7 (1.5)
*C.intermedius*	77∕79∕68∕79	12.8 (0.2)	63.7 (0.8)	19.3 (0.2)	9.8 (1.6)
*C.baileyi*	39∕39∕36∕38	31.5 (0.7)	81.2 (0.7)	26.7 (0.1)	117.0 (2.4)
Cricetidae					
*Peromyscus eremicus*	9∕10∕10∕10	18.0 (0.8)	71.6 (1.5)	19.0 (0.5)	102.0 (4.3)
*Perognathus amplus*	1∕1∕1∕1	12.0	62.0	20.0	82.0
*Neotoma albigula*	18∕21∕20∕21	146.9 (9.4)	144.8 (4.1)	30.8 (0.3)	146.6 (3.2)
Sciuridae					
*Ammospermophilus harrisii*	1∕1∕1∕1	99.0	120.0	36.0	80.0

**Table A2:** Rotated Principal Component Analysis (PCA) of habitat characteristics quantified along mammal trapping transects located at anthropogenic water sources (AWS) and control sites (CS) on Barry M. Goldwater Range in Maricopa County, Arizona, USA. Selections of initial vegetation variables were selected for inclusion in the PCA by variable weight (>0.500). Interpretation of principal components (PC) was based on variables having a high weight for contributing to explaining each component (bolded values).

Habitat characteristics	Component				
	PC1	PC2	PC3	PC4	PC5
Bare ground (% cover)	**−0.984**	0.003	−0.090	−0.041	0.114
Herbaceous (% cover)	**0.984**	−0.001	0.089	0.040	−0.114
Forbs (% cover)	**0.982**	−0.017	0.094	−0.025	−0.043
*L. tridentate* (% cover)	0.003	**0.912**	−0.123	−0.106	−0.253
Shrub (% cover)	−0.122	**0.890**	0.030	−0.091	0.318
*L. tridentate* density/ha.	0.196	**0.613**	−0.473	−0.224	−0.197
Tree density/ha.	0.155	−0.066	**0.892**	0.111	−0.055
Tree (% cover)	0.112	−0.093	**0.883**	0.045	0.108
*C. leptocaulis* density/ha.	0.003	−0.044	0.124	**0.947**	−0.175
Cacti density/ha.	0.053	−0.241	0.071	**0.897**	0.253
Shrub density/ha.	−0.187	−0.035	0.064	0.027	**0.958**
Eigenvalue	3.03	2.07	1.86	1.79	1.26
% Variation explained	27.55	18.85	16.94	16.28	11.45
Cumulative % variation	27.55	46.40	63.34	79.62	91.07

## Additional Information and Declarations

### Field Study Permissions

The following information was supplied relating to field study approvals (i.e., approving body and any reference numbers): Special use permit was issued for access onto the Barry M. Goldwater Range (East) by the 56th Range Management Office of Luke Air Force Base, Arizona.

### Data Availability

The following information was supplied regarding data availability: The raw data has been provided as Supplemental File.
